# Supplementary material for: Ionization Potentials at Mean-Field Computational Cost: The Extended Koopmans’ Framework for pCCD
Source: J Chem Theory Comput. 2026 Mar 13;22(6):2846–60. doi: 10.1021/acs.jctc.5c01922 (PMC13298823; doi:10.1021/acs.jctc.5c01922)
Supplement: Supplementary file 1 [file ct5c01922_si_001.pdf]

Ionization Potentials at Mean-Field Computational Cost: The Extended Koopmans' Framework for pCCD

Seyedehdelaram Jahani,[a] Katharina Boguslawski[a], and Paweł Tecmer,[a*]

[a]*Institute of Physics, Faculty of Physics, Astronomy and Informatics, Nicolaus Copernicus University in Toruń, Toruń, Grudziądzka 5, 87-100 Toruń, Poland*

*Email: k.boguslawski@fizyka.umk.pl; ptecmer@fizyka.umk.pl

Supplementary Information

S1 IP (atoms)

Table S1: Statistical errors w.r.t. experimental IPs from Ref. 1,2 calculated for eight atoms using three different basis sets, including Mean Error (ME), Mean Absolute Error (MAE), Root-Mean-Square Error (RMSE), Mean Percentage Error (MPE), Standard Deviation (SD). The definitions for ME, MAE, RMSE, MPE and SD are ME $= \sum_i^N \frac{E_i^{\text{method}} - E_i^{\text{ref}}}{N}$, MAE $= \sum_i^N \frac{|E_i^{\text{method}} - E_i^{\text{ref}}|}{N}$, RMSE $= \sqrt{\sum_i^N \frac{(E_i^{\text{method}} - E_i^{\text{ref}})^2}{N}}$, MPE $= \frac{1}{N}\sum_i^N \frac{|E_i^{\text{method}} - E_i^{\text{ref}}|}{E_i^{\text{ref}}} \times 100$, SD $= \sqrt{\frac{\sum_i^N \left(E_i^{\text{ME}} - \overline{E_i^{\text{ME}}}\right)^2}{N}}$. Calculations were performed using different convergence thresholds (e.g., (5×10^{-5}), (5×10^{-4}), (1×10^{-3}), and (1×10^{-2})), as indicated in the table.

Method	Basis set	Threshold	ME [eV]	MAE [eV]	RMSE [eV]	MPE [%]	SD [eV]
KT(HF)	cc-pVDZ		−0.27	0.71	0.82	7.11	0.84
	cc-pVTZ	–	−0.19	0.78	0.90	7.48	0.94
	cc-pVQZ		−0.20	0.82	0.94	7.96	0.99
KT(pCCD)	cc-pVDZ		−0.29	0.73	0.86	7.40	0.86
	cc-pVTZ	–	−0.23	0.80	0.93	7.77	0.97
	cc-pVQZ		−0.21	0.82	0.95	7.83	0.99
EKT(HF)	cc-pVDZ	$5e^{-5}$	−2.82	3.09	4.36	36.38	3.56
		$5e^{-4}$	−2.63	2.90	4.00	34.42	3.22
		$1e^{-3}$	−1.12	1.46	2.27	18.86	2.11
		$1e^{-2}$	−0.06	0.39	0.48	3.48	0.51
	cc-pVTZ	$5e^{-5}$	−11.26	11.26	13.61	82.66	8.18
		$5e^{-4}$	−2.01	2.57	3.69	3.69	3.31
		$1e^{-3}$	−1.93	2.64	3.70	31.11	3.37
		$1e^{-2}$	0.20	0.66	0.84	0.84	0.87
	cc-pVQZ	$5e^{-5}$	−17.12	17.12	18.72	132.15	8.08
		$5e^{-4}$	−3.93	4.60	11.33	21.37	11.36
		$1e^{-3}$	0.11	0.64	0.74	5.28	0.78
		$1e^{-3}$	0.17	0.71	0.88	5.60	0.93
EKT(pCCD)	cc-pVDZ	$5e^{-5}$	−0.04	0.31	0.40	2.62	0.42
		$5e^{-4}$	−0.04	0.31	0.39	2.58	0.42
		$1e^{-3}$	−0.04	0.31	0.39	2.58	2.58
		$1e^{-2}$	0.45	0.61	0.80	3.77	0.71
	cc-pVTZ	$5e^{-5}$	0.05	0.38	0.50	3.07	0.54
		$5e^{-4}$	0.11	0.39	0.54	3.02	0.56
		$1e^{-3}$	0.11	0.39	0.54	3.02	0.56
		$1e^{-2}$	0.59	0.75	1.01	4.52	0.87
	cc-pVQZ	$5e^{-5}$	0.11	0.41	0.55	3.29	0.57
		$5e^{-4}$	0.17	0.44	0.58	3.40	0.60
		$1e^{-3}$	0.17	0.44	0.58	3.43	0.60
		$1e^{-2}$	0.62	0.78	1.06	4.66	0.92

S2 IP (acceptor molecules)

Table S2: Ionization potentials (in eV) were determined using various computational schemes with four different basis sets for 24 acceptor molecules taken from the supplementary materials of Refs. 3,4. Experimental values were obtained from Ref. 3, while "Theo." refers to the best theoretical estimates with CCSD(T) level of theory reported in Refs. 3,5. The IP-EOM-pCCD methods are abbreviated as IP-pCCD. Values marked with * indicate IP-pCCD results extrapolated to the basis set limit, as described in Ref. 6.

Molecule	Basis set	Exp.[Ref. 3]	Theo.[Ref. 3,5]	KT[Ref. 7]		EKT		IP-pCCD (2h, 1p)[Ref. 8]		CCSD(T)[Ref. 3]
				HF	pCCD	HF	pCCD	HF	pCCD	HF
Acridine	cc-pVDZ	7.80	8.04	7.59	10.63	7.75	7.88	4.99	5.65	
	cc-pVTZ			7.63		7.79	7.96			
	aug-cc-pVDZ			7.67	10.68	7.79	7.96	4.97	5.69	7.87
	aug-cc-pVTZ			7.67	10.63	7.79	7.99	5.55*		
Anthracene	cc-pVDZ	7.44	7.52	6.96	10.48	7.14	7.36	4.53	5.10	
	cc-pVTZ			6.99		7.17	7.40			
	aug-cc-pVDZ			7.06	10.54	7.17	7.44	4.51	5.14	7.37
	aug-cc-pVTZ			7.03	10.51	7.16	7.42		5.05*	
Azulene	cc-pVDZ	7.42	7.55	7.01	10.10	7.29	7.18	4.65	5.03	
	cc-pVTZ			7.06		7.33	7.26			
	aug-cc-pVDZ			7.10	10.19	7.32	7.29	4.64	5.11	7.38
	aug-cc-pVTZ			7.11	10.15	7.30	7.30		4.97*	
Benzonitrile	cc-pVDZ	9.73	9.93	9.67	11.32	0.80	9.48	7.30	7.62	
	cc-pVTZ			9.73		10.00	9.60			
	aug-cc-pVDZ			9.76	11.39	9.97	9.58	7.22	7.67	9.76
	aug-cc-pVTZ			9.77	11.36	9.94	9.64		7.54*	
Benzoquinone	cc-pVDZ	10.00	10.27	11.15	12.00	11.34	9.39	7.30	9.07	
	cc-pVTZ			11.19		11.40	11.19			
	aug-cc-pVDZ			11.22	12.12	11.34	11.28	7.36	9.20	10.02
	aug-cc-pVTZ			11.23	12.09	11.34	11.26		8.18*	
Dichlone	cc-pVDZ	9.50	9.99	10.06	11.51	10.22	9.90	7.23	7.81	
	cc-pVTZ			10.09		10.22	9.99			
	aug-cc-pVDZ			10.12	11.59	10.24	10.00	7.20	7.80	9.76
	aug-cc-pVTZ			10.11	11.55	10.20	10.02		7.65*	
Furmaronitrile	cc-pVDZ	11.30	11.48	11.19	12.97	−5.28	11.42	9.00	9.63	
	cc-pVTZ			11.28		10.13	11.60			
	aug-cc-pVDZ			11.30	13.09	11.53	11.58	8.94	9.70	11.25
	aug-cc-pVTZ			11.31	13.07	11.54	11.63		9.59*	
Maleic anhydride	cc-pVDZ	11.07	11.33	12.12	12.79	−5.71	11.90	8.22	8.93	
	cc-pVTZ			12.20		12.50	11.98			
	aug-cc-pVDZ			12.23	12.91	12.50	12.11	8.26	9.05	11.05
	aug-cc-pVTZ			12.24	12.90	12.50	12.07		9.05*	
mDCNB	cc-pVDZ	10.20	10.45	10.27	12.02	-5.54	10.03	7.75	8.12	
	cc-pVTZ			10.34		10.57	10.15			
	aug-cc-pVDZ			10.37	12.12	10.56	10.11	7.71	8.17	10.24
	aug-cc-pVTZ			10.38	12.08	10.55	10.19		8.05*	
Napthalenedione	cc-pVDZ	9.50	9.88	9.74	11.21	9.97	9.60	6.84	7.57	
	cc-pVTZ			9.79		10.00	9.70			
	aug-cc-pVDZ			9.82	11.43	9.96	9.62	6.87	7.53	9.65
	aug-cc-pVTZ			9.82	11.27	9.92	9.74		7.38*	
NDCA	cc-pVDZ	8.92	9.14	8.92	11.53	9.09	9.10	6.28	6.85	
	cc-pVTZ			8.96		9.13	9.17			
	aug-cc-pVDZ			8.98	11.60	9.13	9.16	6.24	6.88	8.96
	aug-cc-pVTZ			8.99	11.56	9.13	9.20		6.74*	

Nitrobenzene	cc-pVDZ	9.94	10.19	10.02	11.40	10.31	9.77	7.71	7.97	
	cc-pVTZ			10.04		10.31	9.89			
	aug-cc-pVDZ			10.06	11.45	10.25	9.88	7.60	7.96	10.03
	aug-cc-pVTZ			10.07	11.43	10.23	9.92		7.85*	
Nitrobenzonitrile	cc-pVDZ	10.59	10.62	10.67	12.06	-6.07	10.16	8.00	8.33	
	cc-pVTZ			10.72		10.89	10.31			
	aug-cc-pVDZ			10.74	12.11	10.90	10.31	7.92	8.35	10.03
	aug-cc-pVTZ			10.75	12.09	10.90	10.36		8.28*	
Phenazine	cc-pVDZ	8.44	8.47	8.07	10.84	8.22	8.38	5.43	6.12	
	cc-pVTZ			8.12		8.27	8.46			
	aug-cc-pVDZ			8.15	10.90	8.28	8.44	5.41	6.10	8.31
	aug-cc-pVTZ			8.16	10.87	8.28	8.51		6.02*	
Phthalic anhydrid	cc-pVDZ	10.10	10.55	10.44	11.78	10.72	10.20	7.72	8.25	
	cc-pVTZ			10.47		10.74	10.32			
	aug-cc-pVDZ			10.50	11.86	10.71	10.34	7.75	8.27	10.41
	aug-cc-pVTZ			10.51	11.83	10.68	10.37		8.14*	
Phthalimide	cc-pVDZ	9.90	10.08	10.05	11.42	10.28	9.81	7.08	7.81	
	cc-pVTZ			10.10		10.33	9.91			
	aug-cc-pVDZ			10.14	11.53	10.31	9.91	7.14	7.86	9.91
	aug-cc-pVTZ			10.15	11.51	10.30	9.96		7.73*	
TCNE	cc-pVDZ	11.79	11.99	11.90	14.74	3.24	12.01	9.39	10.04	0
	cc-pVTZ			11.98		12.25	12.19			
	aug-cc-pVDZ			12.00	14.85	12.22	12.17	9.37	10.10	11.74
	aug-cc-pVTZ			12.00	14.82	12.22	12.23		10.00*	
Cl4benzoquinone	cc-pVDZ	9.74	10.25	10.67	13.42	5.25	10.82	7.92	8.58	
	cc-pVTZ			10.65		10.75	10.78			
	aug-cc-pVDZ			10.71	13.50	10.80	10.93	7.83	8.55	9.99
	aug-cc-pVTZ			10.67	13.44	10.76	10.85		8.35*	
Cl4isobenzofuranedione	cc-pVDZ	10.80	10.05	10.43	13.89	10.58	10.30	7.60	7.93	
	cc-pVTZ			10.40		0.88	10.27			
	aug-cc-pVDZ			10.46	13.94	10.61	10.39	7.50	7.89	9.84
	aug-cc-pVTZ			10.42	13.35	10.57	10.32		7.73*	
F4benzenedicarbonitrile	cc-pVDZ	10.65	10.76	11.10	13.63	9.11	10.83	8.14	8.39	
	cc-pVTZ			11.12		11.39	10.98			
	aug-cc-pVDZ			11.18	13.71	11.43	11.05	8.09	8.43	10.52
	aug-cc-pVTZ			11.16	13.70	11.42	11.06		8.27*	
F4benzoquinone	cc-pVDZ	10.70	11.14	11.61	13.55	11.80	9.89	8.66	9.29	
	cc-pVTZ			11.62		11.80	11.90			
	aug-cc-pVDZ			11.69	13.71	11.83	11.94	8.60	9.34	10.89
	aug-cc-pVTZ			11.69	13.71	11.80	11.93		9.17*	
Bodipy	cc-pVDZ	–	8.07	7.69	10.38	8.00	6.85	5.29	5.31	
	cc-pVTZ			7.73		7.97	6.74			
	aug-cc-pVDZ			7.77	10.46	7.95	6.96	5.24	5.37	7.89
	aug-cc-pVTZ			7.78	10.42	7.95	6.79		5.24*	
Dinitrobenzonitrile	cc-pVDZ	–	11.15	11.31	12.89	-6.54	10.86	8.58	8.96	
	cc-pVTZ			11.33		11.50	10.96			
	aug-cc-pVDZ			11.34	13.18	11.48	11.48	8.48	8.95	11.31
	aug-cc-pVTZ			11.34	13.12	11.49	11.00		8.89*	
TCNQ	cc-pVDZ	–	9.57	9.44	12.64	5.71	9.50	6.80	7.39	
	cc-pVTZ			9.54		7.93	9.68			
	aug-cc-pVDZ			9.57	12.75	9.77	9.69	6.83	7.49	9.35
	aug-cc-pVTZ			9.58	12.72	9.78	9.72		7.39*	

S3 XYZ Coordinates in Angstroms (small molecules)

Table S3: CO

Atom	X	Y	Z
C	0.00000	0.00000	0.00000
O	0.00000	0.00000	1.13720

Table S4: N_2

Atom	X	Y	Z
N	0.0000	0.0000	0.0000
N	0.0000	0.0000	1.1136

Table S5: HF

Atom	X	Y	Z
H	0.0000	0.0000	0.0000
F	0.0000	0.0000	0.9178

Table S6: F_2

Atom	X	Y	Z
F	0.0000	0.0000	0.0000
F	0.0000	0.0000	1.4057

Table S7: H_2O

Atom	X	Y	Z
O	0.0000	0.0000	0.0000
H	0.5950	0.7542	0.0000
H	0.5950	−0.7542	0.0000

Table S8: C_2H_4

Atom	X	Y	Z
C	−0.6662	0.0000	0.0000
C	0.6662	0.0000	0.0000
H	−1.2305	0.9251	0.0000
H	−1.2305	−0.9251	0.0000
H	1.2305	0.9251	0.0000
H	1.2305	−0.9251	0.0000

References

[1] P. A. Limacher, *J. Chem. Theory Comput.*, 2015, **11**, 3629–3635.

[2] R. D. Johnson, *NIST Computational Chemistry Comparison and Benchmark Database NIST Standard Reference Database Number 101*, 2013, https://cccbdb.nist.gov/; (accessed October 30, 2025).

[3] R. M. Richard, M. S. Marshall, O. Dolgounitcheva, J. V. Ortiz, J.-L. Bredas, N. Marom and C. D. Sherrill, *J. Chem. Theory Comput.*, 2016, **12**, 595–604.

[4] J. W. Knight, X. Wang, L. Gallandi, O. Dolgounitcheva, X. Ren, J. V. Ortiz, P. Rinke, T. Körzdörfer and N. Marom, *J. Chem. Theory Comput.*, 2016, **12**, 615–626.

[5] A. Shaalan Alag, D. P. Jelenfi, A. Tajti and P. G. Szalay, *J. Chem. Theory Comput.*, 2022, **18**, 6794–6801.

[6] M. Gałyńska, P. Tecmer and K. Boguslawski, *J. Phys. Chem. A*, 2024, **128**, 11068–11073.

[7] S. Jahani, S. Ahmadkhani, K. Boguslawski and P. Tecmer, *J. Chem. Phys.*, 2025, **162**, 184110.

[8] M. Gałyńska and K. Boguslawski, *J. Chem. Theory Comput.*, 2024, **20**, 6174–6186.
